# Circulating cell-free mitochondrial DNA, but not leukocyte mitochondrial DNA copy number, is elevated in major depressive disorder

**DOI:** 10.1038/s41386-017-0001-9

**Published:** 2018-01-30

**Authors:** Daniel Lindqvist, Owen M. Wolkowitz, Martin Picard, Lars Ohlsson, Francesco S. Bersani, Johan Fernström, Åsa Westrin, Christina M. Hough, Jue Lin, Victor I. Reus, Elissa S. Epel, Synthia H. Mellon

**Affiliations:** 1 Faculty of Medicine, Department of Clinical Sciences, Psychiatry, Lund University, Lund, Sweden; 2 Department of Psychiatry, University of California San Francisco (UCSF) School of Medicine, San Francisco, CA USA; 3 Psychiatric Clinic, Lund, Division of Psychiatry, Lund, Sweden; 4 Division of Behavioral Medicine, Department of Psychiatry, Columbia University Medical Center, New York, NY USA; 5 Department of Neurology and Columbia Translational Neuroscience Initiative, Columbia University Medical Center, New York, NY USA; 6 Columbia Aging Center, Columbia University Medical Center, New York, NY USA; 7 Department of Biomedical Science, Malmö University, Malmö, Sweden; 8 Department of Neurology and Psychiatry, Sapienza University of Rome, Rome, Italy; 9 Department of Biochemistry and Biophysics, University of California San Francisco (UCSF) School of Medicine, San Francisco, CA USA; 10 Department of OB/GYN and Reproductive Sciences, University of California San Francisco (UCSF) School of Medicine, San Francisco, CA USA; 11 Present Address: Department of Psychology, University of California, Los Angeles (UCLA), Los Angeles, CA USA

## Abstract

Major depressive disorder (MDD) has been linked to mitochondrial defects, which could manifest in mitochondrial DNA (mtDNA) polymorphisms or mutations. Additionally, copy number of mtDNA (mtDNA-cn) can be quantified in peripheral blood mononuclear cells (PBMC)s, indirectly reflecting cellular energetics, or in the circulating cell-free mtDNA (ccf-mtDNA) levels, which may reflect a fraction of the mitochondrial genome released during cellular stress. Few studies have examined ccf-mtDNA in MDD, and no studies have tested its relationship with intracellular mtDNA-cn or with antidepressant treatment response. Here, mtDNA levels were quantified in parallel from: (i) PBMCs and (ii) cell-free plasma of 50 unmedicated MDD subjects and 55 controls, in parallel with PBMC telomere length (TL) and antioxidant enzyme glutathione peroxidase (GpX) activity. MtDNA measures were repeated in 19 MDD subjects after 8 weeks of open-label SSRI treatment. In analyses adjusted for age, sex, BMI, and smoking, MDD subjects had significantly elevated levels of ccf-mtDNA (*F* = 20.6, *p* = 0.00002). PBMC mtDNA-cn did not differ between groups (*p* > 0.4). In preliminary analyses, we found that changes in ccf-mtDNA with SSRI treatment differed between SSRI responders and non-responders (*F* = 6.47, *p* = 0.02), with the non-responders showing an increase in ccf-mtDNA and responders not changing. Baseline ccf-mtDNA was positively correlated with GpX (*r* = 0.32, *p* = 0.001), and PBMC mtDNA correlated positively with PBMC TL (*r* = 0.38, *p* = 0.0001). These data suggest that plasma ccf-mtDNA and PBMC mtDNA-cn reflect different cellular processes and that the former may be more reflective of certain aspects of MDD pathophysiology and of the response to SSRI antidepressants.

## Introduction

Mitochondrial dysfunction may be involved in the pathophysiology of MDD [1–5]. Cells contain multiple mitochondria and, in turn, each mitochondrion contains multiple copies of its own genome, the mitochondrial DNA (mtDNA), which encodes 37 genes essential to energy production [6]. Mitochondria may be conceptualized as the “power generators” of the cell, converting oxygen, energy substrates (proteins, carbohydrates and lipids) and other substrates into adenosine triphosphate [7]. Beyond energy production, mitochondria generate other signaling molecules that influence cellular and physiological functions, [8, 9] including cellular senescence [10] and production of free radicals [11]. As a result of these mechanisms, mitochondrial impairments may be associated with, and/or lead to, psychiatric manifestations such as mood and anxiety disorders, conditions that have been associated with cellular senescence and oxidative stress. [12, 13]

mtDNA copy number (mtDNA-cn), which represents the number of mitochondrial genomes per cell [6], can be quantified in peripheral blood mononuclear cells (PBMC) and is thought to reflect variations in mitochondrial energetic function and biogenesis [6]. Additionally, mtDNA may be released at low levels into the circulation from mitochondria under cellular stress, resulting in circulating cell-free mtDNA (ccf-mtDNA) detectable in plasma [14, 15]. Ccf-mtDNA has only very recently been assessed in psychiatric patients after suicide attempt [16], and in MDD subjects [17].

Although the exact source or significance of ccf-mtDNA in psychiatric illness is yet to be determined, it is suggested to reflect cellular extrusion of mitochondria, during apoptotic or necrotic cell death or other processes, resulting in mtDNA appearing in the plasma [18, 19]. In addition, oxidative stress may promote the release of mtDNA into the cell cytoplasm, and possibly into the extracellular space [20]. Chronic neuroendocrine, inflammatory, oxidative and metabolic stress may result in mitochondrial dysfunction and mtDNA damage [21]. Downstream effects of this stress–disease cascade involve cellular dysfunction and senescence, and the release of ccf-mtDNA into the bloodstream, which in turn may have detrimental effects on multiple organ systems via inflammatory mechanisms [21, 22]. Although this has not been well studied in psychiatric settings, increased blood levels of free-circulating mtDNA have been reported in a number of different somatic conditions such as diabetes [23], cancers [15], and myocardial infarction [24] and have been linked to more severe illness and worse outcome [25].

Levels of ccf-mtDNA and cellular mtDNA-cn may have further significance, as they also relate to cellular oxidative stress and cellular senescence. MtDNA is highly vulnerable to oxidative stress due to the lack of protective histones and limited DNA repair mechanisms [26]. Antioxidant enzymes such as glutathione peroxidase (GpX) are therefore needed in order to protect the mitochondria and prevent cellular dysfunction [27]. Mitochondrial integrity and bioenergetics are also related to nuclear DNA senescence, due to a direct link between telomere shortening, repression of peroxisome proliferator-activated receptor gamma coactivator 1-alpha (PGC-1α) and diminished mitochondrial biogenesis in certain tissues [28]. Thus, assessment of mtDNA, in the cell-free circulation or in the intracellular compartment may also inform on cellular stress and cellular senescence [21], although the potential relevance of these interactions for MDD is currently unclear.

Despite emerging evidence for a link between high mtDNA and MDD [5, 29], there are inconsistent results across studies, [30–32] and several unresolved issues remain. These include the extent to which different measurements of mtDNA (e.g., leukocyte mtDNA-cn vs. ccf- mtDNA) inter-correlate in subjects with and without psychopathology, and how mtDNA levels in different compartments relate to other biological markers implicated in MDD, such as oxidative stress and indices of accelerated cellular aging. Furthermore, most previous studies in MDD have included subjects taking antidepressants or mood stabilizers, [5,29–31,^33^] highlighting the importance of studying this in well-characterized  unmedicated MDD samples. Moreover, no previous studies have investigated the relationship between leukocyte mtDNA-cn or ccf-mtDNA and antidepressant treatment response. The purposes of the present study were, therefore, to (i) investigate cellular and circulating cell-free levels of mtDNA in a sample of well-characterized, unmedicated, and somatically healthy MDD subjects and healthy controls, (ii) test the relationship between mtDNA and peripheral indices of oxidative stress and accelerated cellular aging (telomere length), and (iii) determine if successful antidepressant treatment is associated with changes in cellular mtDNA-cn or ccf- mtDNA.

## Methods and materials

### Ethics statement

The Committee on Human Research of the University of California, San Francisco (UCSF) approved the study protocol. All study participants gave written informed consent to participate in this study and were compensated for participating.

### Baseline recruitment procedures and study participants

MDD (*n* = 50) outpatients and healthy controls (*n* = 55) were recruited between 2011–2015 by flyers, bulletin board notices, Craigslist postings, newspaper ads and, in the case of MDD subjects, clinical referrals. Ccf-mtDNA measurements were missing for two subjects, and PMBC mtDNA-cn was missing from two subjects. All diagnoses, including MDD, were established using the structured clinical interview [34] and were verified in a separate diagnostic evaluation by a Board-certified psychiatrist. Depression symptom severity was assessed in MDD subjects using the Hamilton Depression Rating Scale (HDRS) [35]. All MDD subjects had a minimum 17-item HDRS [35] score of 17. MDD subjects were excluded if they met DSM-IV criteria (which were the extant criteria in use at the time this study was conducted) for any of the following: (i) bipolar disorder, (ii) alcohol or substance abuse within the preceding 6 months, (iii) PTSD or an eating disorder within 1 month of entering the study, and (iv) for any history of psychosis outside of a major depressive episode (MDE), or the presence of any psychotic symptoms during the current MDE. Potential healthy controls were excluded for any history of DSM-IV Axis-I diagnoses. All study participants were free of acute illnesses or infections, inflammatory disorders, neurological disorders, or any other medical conditions considered to be potentially confounding (e.g., cancer, HIV, diabetes, history of cardiovascular disease or stroke, etc.), as assessed by history, physical examinations, and routine blood screening. All subjects were free of psychotropic medications (including antidepressants), hormone supplements, steroid-containing birth control or other potentially interfering medications, including vitamin supplements above the U.S. recommended daily allowances (e.g. >90 mg/day for Vitamin C) and had not had any vaccinations for at least 6 weeks prior to enrollment in the study. For the MDD subjects, short-acting sedative-hypnotics were allowed as needed up to a maximum of three times per week, but none within 1 week prior to blood draws in the study. All subjects had to pass a urine toxicology screen for drugs of abuse (marijuana, cocaine, amphetamines, PCP, opiates, methamphetamine, tricyclic antidepressants, and barbiturates) and a urine test for pregnancy in women of child-bearing age on the same day as the blood draw.

### Selective serotonin reuptake inhibitor (SSRI)-treatment

Nineteen MDD subjects underwent 8 weeks of protocol-based open-label outpatient treatment with an SSRI antidepressant (NCT00285935, https://www.clinicaltrials.gov/). In order to limit the range of potential mechanism of action of antidepressants, the choice of medication was limited to an SSRI. The decision for the specific SSRI prescribed was based on clinical information such as medical history, family history, prior medication history, subject preference and potential side effects. Compliance and clinical evaluations and assessments of drug tolerability were assessed by a telephone check-in at the end of week 1 and an in-person check-in at the end of week 4 and week 8, at which times pill counts were performed. Plasma SSRI concentrations were assessed at the week 4 and week 8 visits to evaluate medication compliance. Primary outcome measure was severity of depressive symptoms, assessed by means of the HDRS. HDRS ratings were repeated at the end of treatment (week 8). Twelve subjects were treated with sertraline (mean ± SD dose in mg = 142 ± 36), two with fluoxetine (mean ± SD dose in mg = 35 ± 7), two with citalopram (mean ± SD dose in mg = 35 ± 7), and three with escitalopram (mean ± SD dose in mg = 13 ± 6). Medication dosages were increased over the course of treatment per pre-specified protocol as tolerated and as warranted by clinical response. Sertraline dosing began with 25 mg per day and increased to a maximum of 200 mg per day; fluoxetine and citalopram dosing began with 10 mg per day and increased to a maximum of 40 mg per day; escitalopram dosing began with 10 mg per day and increased to a maximum of 20 mg per day. “Responders” were defined as subjects with greater than or equal to 50% improvement on HDRS ratings at week 8 compared to baseline. There was no significant difference in final SSRI dose (sertraline equivalents) between responders and non-responders (*p* = 0.59), and all subjects (responders and non-responders) had plasma SSRI levels within expected reference ranges.

### Blood sampling and DNA extraction

Venipuncture was performed at approximately 10:00 am at the UCSF Clinical and Translational Science Institute, after 12 h of fasting (except water). Plasma was collected with a lavender EDTA vacutainer tube. Tubes were spun at 1500×*g* for 10 min and 4° C, and plasma was removed, aliquoted into tubes and frozen at −80° C until used. PBMCs were prepared from whole blood by ficoll centrifugation [36] and were frozen at −80 °C. DNA was extracted from frozen PBMCs using commercially available reagents (Puregene, Gentra Systems, Qiagen, Valencia, CA). DNA quality and quantity were assessed using Nanodrop spectrophotometer and random samples were also assessed by agarose gel electrophoresis to assess DNA integrity.

### Measurement of mtDNA-cn in PBMCs

The mtDNA-cn in PBMCs was determined by multiplex real-time polymerase chain reaction (PCR) assay simultaneously quantifying genomic mitochondrial (ND1) and nuclear (Ribonuclease P) amplicons, as described elsewhere [37]. Lab personnel who performed the assay were blind to demographic and clinical data.

### Measurement of ccf-mtDNA in plasma

The isolation and quantification of mtDNA in plasma samples has been previously described [16]. Briefly, the thawed plasma samples were centrifuged for 10 min at 10,000×*g* to remove cells and cellular debris.

DNA were isolated from 200 μl of the supernatant using the QIAmp 96 DNA Blood Kit (Qiagen, Valencia, CA, USA) according to the manufacturer’s instruction for blood and body-fluid protocol. The isolated DNA was then eluted in 200 μl and quantified using spectrophotometric analysis at 260/280 nm in a Nanodrop (ND-1000 Spectrophotometer v 3.7.1, Waltham, MA, USA). The quantitative analysis of ccf-mtDNA was performed using quantitative real time polymerase chain reaction. The experiment was run once in triplicate reactions. A dilution series consisting of the purified PCR product from a healthy control subject, not taking part in the present study, was constructed and used to create a standard curve. The DNA sequence of the PCR-fragment was determined before the dilution series was carried out. The different crossing-point values from the unknown samples were compared with the standard curve, and the corresponding number of mitochondrial units was calculated. The amount of DNA (g μl^−1^) was divided with the size of the PCR-fragment (161 bp) and the molar mass per base pair (g mol^−1^). The product was finally multiplied with Avogadro’s constant.

Mitochondrially encoded NADH: Ubiquinone Oxidoreductase Core Subunit 2 (MT-ND2) gene was amplified using the following primers (Life Technologies, Pailsey, UK): Forward primer: [CACACTCATCACAGCGCTAA]; reverse primer: [GGATTATGGATGCGGTTGCT]. In order to verify the data, the assay was also completed with another target, the mtDNA-encoded NADH: Ubiquinone Oxidoreductase Core Subunit 1 (MT-ND1) gene. This gene was amplified in the same way as above, but using the following primers (Life Technologies, Paisley, UK): Forward primer: [CCCTAAAACCCGCCACATCT]; reverse primer: [CCGATCAGGGCGTAGTTTGA]. Both amplicons were strongly correlated ( *r*= 0.73, *p* < 0.001), indicating that inter-individual mtDNA sequence variation did not affect our ability to detect ccf-mtDNA levels.

The PCR reactions were carried out using SYBR Green Technology (Thermo Fisher Scientific, Waltham, MA, USA). Each 20 μl reaction contained 5 μl of template, 1 μl of each primer (10 μM), 10 μl SYBR MIX (2 × Sensifast, Bioline, London, UK) and 3 μl of nuclease-free water. Each reaction was run in triplicate on a LC480 (LightCycler from Roche, Mannheim, Germany) using the following program:

Initial denaturation at 95 °C for 10 min, followed by 45 cycles consisting of 95 °C in 10 s. For melting, 65 °C for 10 s annealing and 72 °C for 11 s extension. The program ended with a melting curve analysis measuring fluorescence continuously from 60 to 97 °C.

### Measurement of GpX

Glutathione peroxidase activity (BioVision, Inc., Milpitas, California, USA) was measured in duplicate from plasma, using a colorimetric assay according to the instructions from the manufacturer. The coefficient of variation was < 10% and LLOQ was 0.5 nmol NADPH/ml/min.

### Measurement of PBMC telomere length

The telomere length measurement assay is adapted from the published original method by Cawthon [38, 39].

The telomere thermal cycling profile consists of:

Cycling for T(telomic) PCR: 96 °C for 1 min; denature at 96 °C for 1 s, anneal/extend at 54 °C for 60 s, with fluorescence data collection, 30 cycles.

Cycling for S (single copy gene) PCR: PCR: 96 °C for 1 min; denature at 95 °C for 15 s, anneal at 58 °C for 1 s, extend at 72 °C for 20 s, 8 cycles; followed by denature at 96 °C for 1 s, anneal at 58 °C for 1 s, extend at 72 °C for 20 s, hold at 83 °C for 5 s with data collection, 35 cycles.

The primers for the telomere PCR are *tel1b* [5′-CGGTTT(GTTTGG)_5_GTT-3′], used at a final concentration of 100 nM, and *tel2b* [5′-GGCTTG(CCTTAC)_5_CCT-3′], used at a final concentration of 900 nM. The primers for the single-copy gene (human beta-globin) PCR are *hbg1* [5′-GCTTCTGACACAACTGTGTTCACTAGC- 3′], used at a final concentration of 300 nM, and *hbg2* [5′-CACCAACTTCATCCACGTTCACC-3′], used at a final concentration of 700 nM. The final reaction mix contains 20 mM Tris-HCl, pH 8.4; 50 mM KCl; 200 mM each dNTP; 1% DMSO; 0.4× Syber Green I; 22 ng E. coli DNA per reaction; 0.4 Units of Platinum Taq DNA polymerase (Invitrogen Inc.) per 11 µl reaction; ~6 ng of genomic DNA. Tubes containing 26, 8.75, 2.9, 0.97, 0.324 and 0.108 ng of a reference DNA (pooled human genomic DNA from leukocytest) are included in each PCR run so that the quantity of targeted templates in each research sample can be determined relative to the reference DNA sample by the standard curve method. The same reference DNA will be used for all PCR runs.

To control for inter-assay variability, eight control DNA samples are included in each run. In each batch, the T/S ratio of each control DNA is divided by the average T/S for the same DNA from 10 runs to get a normalizing factor. This is done for all eight samples and the average normalizing factor for all eight samples is used to correct the participant DNA samples to get the final T/S ratio. The T/S ratio for each sample will be measured twice. When the duplicate T/S value and the initial value vary by more than 7%, the sample will be run the third time and the two closest values were reported.

### Statistical analysis

The Statistical Package for the Social Sciences (SPSS) v.22 (IBM Corp., Armonk, NY) was used for statistical calculations. All tests were two-tailed with an alpha = 0.05. Non-normally distributed variables were log-transformed to achieve normality, as was the case for both ccf-mtDNA and PBMC mtDNA-cn. In cases when log transformation was insufficient (viz., baseline HDRS scores), we used Blom transformation, a statistical procedure replacing each raw score with its rank value and adjusting the scale distances between the ranks to achieve a normal distribution. As GpX was assayed in two separate batches, we combined *z*-scores from both batches into one variable for analyses. Correlations were tested using Pearson’s *r*, adjusting for covariates using partial correlations. Group comparisons are presented as unadjusted (Student’s *t*-test) and adjusted (ANCOVA) analyses. Repeated measures ANOVAs were used to test the time x group interaction on the changes in mtDNA between baseline and week 8 in SSRI responders and non-responders. Covariates were age, sex, body mass index (BMI) and smoking status, analyses are presented as unadjusted and adjusted.

## Results

### Demographic characteristics

Demographic characteristics of MDD subjects and controls and of SSRI responders and non-responders are presented in Table 1. There were no significant between-group differences with regard to age, and sex. Compared to controls subjects, MDD subjects had higher BMI, and were more likely to be current tobacco users. SSRI non-responders had higher BMI than SSRI responders (*p* = 0.054). After 8 weeks of SSRI treatment, HDRS score was 6.8 ± 2.4 (mean ± SD) for responders and 14.6 ± 4.8 (mean ± SD) for non-responders. For five subjects, this was the first depressive episode, and all other subjects had suffered from previous depressive episodes (median total number of depressive episodes = 4).

**Table 1:** Demographic and clinical characteristics of subjects with MDD and controls and in SSRI responders and non-responders

	Controls *N* = 55	MDD *N* = 50	*P*-value	Responders *N* = 11	Non-responders *N* = 8	*P*-value
Sex (f/m)	33/22	27/23	0.54	8/3	5/3	0.64
Age (Years; mean ± SD)	37.6 ± 13.9	39.6 ± 14.7	0.47	39.2 ± 12.6	39.0 ± 14.9	0.98
BMI (kg/m^2^; mean ± SD)	24.4 ± 4.9	26.1 ± 4.5	0.07	26.2 ± 5.4	30.1 ± 2.7	0.05
Current tobacco users (*n*)	3	13	< 0.01	3	2	0.91
HDRS score at baseline (mean ± SD)	N/A	20.2 ± 3.3	N/A	19.8 ± 3.0	19.6 ± 4.0	0.91

*HDRS* Hamilton Depression Ratting Scale, *BMI* body mass index

### MtDNA at baseline in MDD subjects vs. controls, and correlations with depression symptom severity ratings

**Fig. 1:**
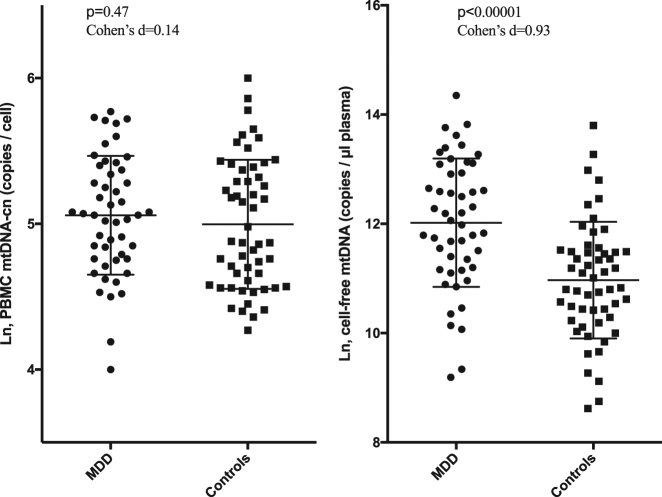
Circulating cell-free mitochondrial DNA (Ccf-mtDNA) and peripheral blood mononuclear cell (PBMC) mtDNA copy number in MDD subjects and controls. Data were log-transformed. Error bars indicate ±1 SD

MDD subjects had significantly higher levels of ccf-mtDNA compared to healthy controls (mtDNA copies per µl plasma, log-transformed, mean ± SD: 12.0 ± 1.2 vs. 11.0 ± 1.1, *t* = 4.8, *p* < 0.00001, Cohen’s *d* = 0.93). MDD subjects did not differ significantly from controls in mtDNA-cn measured in PBMCs (mtDNA copies per cell, log-transformed, mean ± SD: 5.1 ± 0.4 vs. 5.0 ± 0.4, *t* = 0.7, *p* = 0.48, Cohen’s *d* = 0.14). After adjusting for age, sex, BMI and smoking, group difference in cell-free plasma mtDNA remained significant (*F* = 20.6, *p* = 0.00002), while PMBC mtDNA-cn remained non significant (*F* = 0.2, p = 0.65). Baseline mtDNA measurements (ccf-mtDNA and PBMCs) in MDD subjects and controls are shown in Fig. 1. In order to verify these data, we also an analyzed ccf-MtDNA using another target (MT-ND1). This yielded similar results and confirmed the difference in ccf-mtDNA levels between MDD subjects and controls (*p* < 0.001, Cohen’s *d* = 0.78).

Within the MDD subjects there were no significant correlations between HDRS scores at baseline and mtDNA levels in cell-free plasma (*r* = 0.03, p = 0.86), or PBMC (*r* = −0.27, *p* = 0.064). Number of depressive episodes did not correlate significantly with either PBMC mtDNA-cn (*r* = 0.21, *p* = 0.14) or plasma ccf-mtDNA (*r* = −0.12, *p* = 0.42).

### MtDNA levels after 8 weeks of SSRI treatment in a subsample of MDD subjects, preliminary analyses

**Fig. 2:**
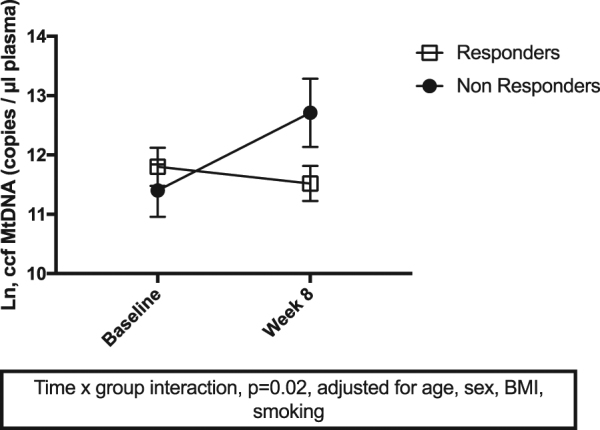
Change in circulating cell-free mitochondrial DNA (ccf-mtDNA) (mean + SEM) between baseline and week 8 in SSRI responders and non-responders. Data were log-transformed

Ccf (*n* = 19) and PBMC (*n* = 18) mtDNA were available from a subset of MDD subjects before and after 8 weeks of SSRI treatment, as described above. MtDNA data from healthy controls were only available at baseline. A significant time (baseline vs. week 8) × group (SSRI responders vs. non-responders) interaction was observed for ccf-mtDNA (unadjusted *F* = (1,17) 4.43, *p* = 0.05; adjusted *F* = (1,13) 6.47, *p* = 0.02), but not for PBMC mtDNA-cn (all *p* > 0.4). After Bonferroni correction, non-responders showed a significant increase in ccf-mtDNA between baseline and week 8 (unadjusted *p* = 0.04; adjusted *p* = 0.02), while this was not seen in responders (all *p* > 0.32). In cross-sectional analyses, ccf-mtDNA was higher in non-responders compared to responders at week 8 at trend level (unadjusted *p* = 0.06; adjusted *p* = 0.08), but did not significantly differ at baseline (all *p* > 0.13). Further, SSRI non-responders had significantly higher ccf-mtDNA levels at week 8 compared to healthy control levels at baseline (*p* < 0.001), whereas no significant difference was seen between SSRI responders at week 8 and healthy controls at baseline (*p* = 0.37). Adjusting for age, sex, BMI and smoking did not substantially alter these findings. Change in ccf-mtDNA between baseline and week 8 in responders and non-responders is shown in Fig. 2.

At week 8, there were no significant correlations between HDRS scores and plasma cell-free mtDNA levels, or in PBMC mtDNA-cn (all *p* > 0.2). Absolute change in HDRS scores were not correlated with absolute change in either ccf-mtDNA or PBMC mtDNA-cn (all *p* > 0.1)

### Correlations between mtDNA and other biomarkers

Across all subjects, ccf-mtDNA and PBMC mtDNA-cn measurements were not inter-correlated (unadjusted: *r* = −0.02, *p* = 0.87; adjusted: *r* = −0.03, *p* = 0.79).

Across all subjects, Ccf-mtDNA, but not PBMC mtDNA-cn, was positively correlated with antioxidant enzyme glutathione peroxidase (ccf-mtDNA unadjusted and adjusted: *r* = 0.32, *p* = 0.001; PBMC mtDNA-cn unadjusted and adjusted: *r* = −0.12, *p* = 0.26). Conversely, PBMC mtDNA-cn, but not ccf-mtDNA, was positively correlated with PBMC telomere length (PBMC mtDNA-cn unadjusted: *r* = 0.28, *p* = 0.005, adjusted: *r* = 0.38, *p* = 0.0001; ccf-mtDNA: unadjusted: *r* = 0.08, *p* = 0.42, adjusted: *r* = 0.11, *p* = 0.27).

Overall, these correlations indicate that about 10% in the variance of ccf-mtDNA and PBMC mtDNA-cn (bivariate correlations) is accounted for by differences in antioxidant activity and telomere length, respectively.

**Fig. 3:**
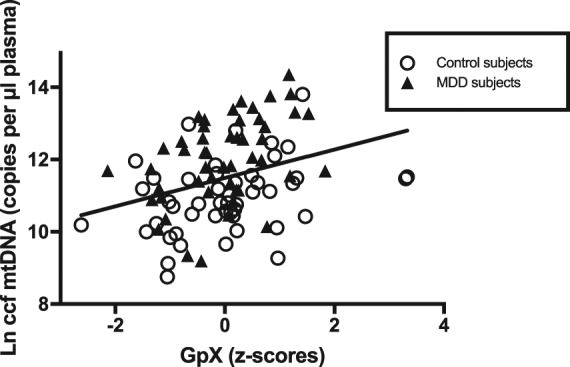
Circulating cell-free mitochondrial DNA (ccf mtDNA) (log-transformed) plotted against glutathione peroxidase activity (GpX) (*z*-scores) in MDD subjects and healthy controls. Pearson’s *r* = 0.32, *p* = 0.001

**Fig. 4:**
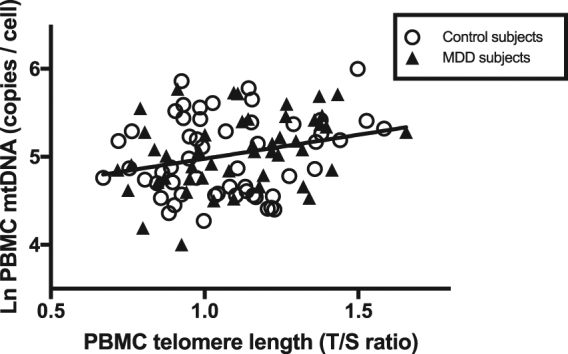
Peripheral blood mononuclear cell (PBMC) telomere length plotted against PBMC mitochondrial DNA-copy number (mtDN-cn) (log-transformed) in MDD subjects and healthy controls. Pearson’s *r* = 0.28, *p* = 0.005

Figs. 3 and 4 show these correlations in MDD and control subjects separately.

## Discussion

Our results show that ccf-mtDNA is significantly higher in un-medicated MDD subjects compared to healthy controls, but that cellular (PBMC) mtDNA-cn does not significantly differ between groups. PBMC mtDNA-cn likely reflects intracellular mtDNA content and bioenergetics [6], independent of apoptosis; however, ccf-mtDNA is likely released from cells during cellular stress [19, 40] and does not contribute to cell energetics. Consistent with the idea that they may reflect different processes, ccf-mtDNA and cellular PBMC measures of mtDNA-cn were not significantly correlated. Additionally, the present study found that ccf-mtDNA levels further increased in non-responders to SSRI treatment over the 8-week course of treatment, whereas ccf-mtDNA levels in responders did not significantly change. After 8 weeks of SSRI treatment, ccf-mtDNA remained significantly elevated in non-responders compared to baseline levels of controls, whereas, at the same time point, there was no significant difference between responders and baseline levels of controls. In contrast, cellular (PMBC) mtDNA-cn did not significantly change over the course of SSRI treatment in either responders or non-responders. These data suggest that ccf-mtDNA and cellular PBMC mtDNA-cn reflect different cellular processes and that the former may be more reflective of certain aspects of MDD pathophysiology and of the response to SSRI antidepressants.

Studies of leukocyte mtDNA-cn in MDD have yielded inconsistent results, and only two previous studies have examined ccf-mtDNA in psychiatric samples. In the largest study in MDD to date, Cai et al. showed that greater mtDNA-cn in saliva and in blood leukocytes was associated with MDD [5]. The same group later confirmed, in a longitudinal study, that MDD subjects have higher cellular mtDNA-cn compared to controls over 8 weeks of antidepressant treatment. Across all time points, but not cross-sectionally, mtDNA-cn was positively correlated with depression severity [29]. Other studies have, however, reported lower [30] or unchanged [31] leukocyte mtDNA-cn in MDD compared to controls. Moreover, two population-based studies reported negative correlations between leukocyte mtDNA-cn and depressive symptoms [41, 42], while a recent longitudinal study did not find any between-person or within-person associations between depressive symptoms and leukocyte mtDNA-cn [32]. In regards to other affective disorders, one study on euthymic bipolar disorders showed lower leukocyte mtDNA-cn compared to controls [33], while another study on subjects with bipolar disorder in a depressive episode did not find a significant difference in mtDNA-cn compared to controls, either before or after lithium treatment [43]. Reasons for the discrepancy in findings in these studies of cellular mtDNA-cn are not known, but could involve medication status of the subjects, different sample sizes across studies, somatic co-morbidities, differences in age, ethnicity or gender composition, differences in phenotypes (e.g., MDD diagnosis vs. depressive symptoms) or methodological differences (e.g., whole blood vs. isolated PBMCs). We note that our study might have been underpowered to detect a significant relationship between cellular, PBMC, mtDNA-cn and an MDD diagnosis, since some previous studies demonstrating such an effect had larger samples sizes [5, 29].

Only two studies have previously examined ccf-mtDNA in a psychiatric population. Our research group previously reported that ccf-mtDNA is strongly increased (Cohen’s *d* = 2.55–4.01) in suicidal individuals (whether or not with MDD diagnoses) compared to healthy controls [16]. The present study only included subjects with MDD, and none were actively suicidal. In contrast to our study, Kageyama et al. recently reported lower ccf-mtDNA levels in MDD subjects compared to controls [17]. Differences in subject recruitment, blood sampling and statistical modeling may have contributed to the divergent findings. For example, Kageyama et al. did not actively exclude subjects with potentially confounding medical conditions, and they did not adjust for the effects of smoking or BMI on mtDNA.

Whereas cellular mtDNA-cn is a marker of mitochondrial biogenesis and energetics [6], ccf-mtDNA is believed to be released during cellular injury [19, 40]. Ccf-mtDNA molecules are subsets of so called damage-associated molecular patterns, which may have detrimental downstream effects involving immune activation [22]. Consistent with their reflecting different aspects of mitochondrial/cellular physiology, ccf-mtDNA was not significantly correlated with cellular (PBMC) mtDNA-cn in our study. The differential significant correlations we observed between ccf-mtDNA and PBMC mtDNA-cn versus antioxidant enzyme activity and telomere length, respectively, are also consistent with these functional differences.

During states of increased cellular and oxidative stress, genes encoding antioxidant enzymes, such as GpX, may increase their expression in an attempt to restore cellular homeostasis [44]. Oxidative stress can damage cellular integrity and, if unchecked, can lead to cellular endangerment or apoptosis, both of which can lead to mtDNA extrusion from the cell or release into the blood, increasing ccf-mtDNA. In fact, infusions of the anti-oxidant Vitamin C were observed to diminish ccf-mtDNA in Intensive Care Unit patients [45]. We had predicted an inverse relationship between GPx levels and ccf-mtDNA, although the opposite was found. Although speculative and in need of replication, our finding of a direct correlation between ccf-mtDNA levels and GpX activity may reflect a compensatory attempt to, ineffectively, upregulate the body’s antioxidant defense mechanisms due to cellular stress. This hypothesis is in line with several preclinical studies reporting that GpX may protect against apoptotic cell death [44] and oxidative stress induced mtDNA damage [27].

Telomere shortening can reciprocally lead to cellular mitochondrial endangerment and diminished mitochondrial biogenesis via diminution of PGC-1α, the master regulator of mitochondrial biogenesis [28]. Telomeres become shorter due to each cell division, DNA damage, and exposure to oxidative stress [13]. In our study, PBMC mtDNA-cn was correlated with PBMC telomere length, a marker of cellular aging. This is in line with several previous studies reporting a positive association between mtDNA (measured in leukocytes) and telomere length [32, 41, 46, 47].

Oxidative stress and telomere shortening can both be induced by mitochondrial dysfunction, or mitochondrial allostatic load [21]. Although mitochondrial function was not measured directly here, the present data suggest that ccf-mtDNA may represent a more sensitive marker of cellular damage or cellular stress, not reflective of cellular mtDNA content or cellular energetics. The underlying mechanisms behind cellular stress and mitochondrial dysfunction in mood disorders are not yet fully understood, but might involve a wide array of pathophysiological processes. For instance, elevated stress and increased levels of pro-inflammatory cytokines may lead to accumulation of tryptophan catabolites (TRYCATS) via activation of the kynurenine pathway. TRYCATS may alter mitochondrial function both directly and via the metabolism of the endogenous antioxidant melatonin [48], with potential relevance for the pathophysiology of MDD.

In several of the previous studies investigating the relationship between mtDNA and depressive symptoms, a high proportion of depressed subjects was using antidepressant medications [5, 30, 31, 41, 42]. The participants in our study were free of any psychiatric medication for a period of at least 6 weeks prior to enrollment. This is important since experimental studies have shown that antidepressants may cause alterations in mitochondrial function [49], e.g., via inhibition of mitochondrial enzyme complexes [50], and uncoupling of oxidative phosphorylation [51]. Data from experimental studies suggest that antidepressants may have both pro-apoptotic [52], and anti-apoptotic properties [53], and that some of these effects may be mediated via mitochondria-associated pathways [54]. Interestingly, Djordejevic et al. found that fluoxetine induced apoptosis in rat liver cells, but these alterations were more pronounced in stressed animals compared to non-stressed animals [52]. Based on these data, we speculate that antidepressants may have pro-apoptotic or anti-apoptotic effects depending on symptom trajectory during the treatment course. This is in line with our observation that ccf-mtDNA continued to be higher in those subjects who did not respond to SSRI treatment, whereas there was no difference in ccf-mtDNA between SSRI responders and healthy controls. We note, however, that there was no significant difference in ccf-mtDNA between SSRI responders and non-responders at baseline. Thus, our data do not support the usefulness of ccf-mtDNA as a predictor of antidepressant treatment response, although larger samples are needed to draw any firm conclusions about this.

Strengths of the present study include (i) the simultaneous assessment of ccf-mtDNA as well as PBMC mtDNA-cn, (ii) the use of physically healthy and well-characterized subjects, (iii) the minimum 6-week medication-free period, and (iv) the exclusion of concomitant medications that could influence our results. Further, this is among the first studies to measure mtDNA, either cellular or cell-free plasma, before and after antidepressant treatment. However, the relatively small number of subjects receiving antidepressants yielded low power to test the relationship between ccf-mtDNA, cellular mtDNA-cn and SSRI response. Therefore, our results pertaining to the association between mtDNA levels and SSRI treatment response should be considered preliminary and in need of replication in larger samples. There are also several methodological factors that could have influenced our results. For example, the apparent increase of mtDNA in MDD subjects could be secondary to platelet or leukocyte lysis during the blood sampling, leading to subsequent release of mtDNA into the plasma. However, the blood sampling procedures for MDD subjects and controls was identical, so we believe that it is very unlikely that such methodological factors may have confounded our findings. Finally, another important limitation of our study is that mtDNA was measured in the peripheral blood, thus we were not able to determine if these biological markers are related to brain pathology in MDD. This would be an important area of future studies since we are not aware of any previous reports testing the relationship between peripheral measurements of mtDNA and brain imaging indices. Interestingly though, one recent post-mortem study [55] found alterations in mtDNA in the dorso-lateral prefrontal cortex of suicide completers, suggesting that changes in mtDNA might be relevant also for brain pathology.

In conclusion, our results indicate that ccf-mtDNA is a physiologically distinct marker from cellular (PBMC) mtDNA-cn. Even after adjustment for potential confounders, ccf-mtDNA was elevated in un-medicated MDD and continued to increase in subjects refractory to an 8-week SSRI treatment, whereas patients whose depressive symptoms substantively decreased post-treatment did not demonstrate changes in ccf-mtDNA levels. Together, these data suggest that elevated ccf-mtDNA may be a novel biological correlate of MDD and responsive to treatment, although the possible mechanistic relationship of this marker to depressive pathophysiology, and whether this is a state- or trait-related marker, remain unknown. Given previous inconsistencies in reported associations between intracellular leukocyte mtDNA-cn and depression, our findings point to mtDNA in cell-free plasma as a promising biological correlate of depression. The specificity of these findings for MDD is also unknown and will require further testing.

## Electronic supplementary material

CONSORT flowchart
