# Supplementary material for: Circulating cell-free mitochondrial DNA, but not leukocyte mitochondrial DNA copy number, is elevated in major depressive disorder
Source: Neuropsychopharmacology. 2018 Jan 30;43(7):1557–64. doi: 10.1038/s41386-017-0001-9 (PMC5983469; doi:10.1038/s41386-017-0001-9)
Supplement: Supplementary file 1 — CONSORT flowchart [file 41386_2017_1_MOESM1_ESM.doc]

Assessed for eligibility (n= 149, 84 MDD & 65 HC)

Excluded (n= 44, 34 MDD & 10 HC)

♦  Not meeting inclusion criteria (n=34, 27 MDD & 7 HC)

♦  Declined to participate (n=8, 6 MDD & 2 HC)

♦  No blood samples available for mtDNA analysis (n=2, 1 MDD & 1 HC)

Received SSRI for 8 weeks and had ratings and venipuncture at week 8 (n=19, all MDD)

Were not offered treatment (n=55 HC, N=26 MDD) and had only baseline data

♦ No venipuncture (n= 5, all MDD)

Enrolled at baseline (n= 105, 50 MDD & 55 HC)
